# The Analysis of a Microbial Community in the UV/O_3_-Anaerobic/Aerobic Integrated Process for Petrochemical Nanofiltration Concentrate (NFC) Treatment by 454-Pyrosequencing

**DOI:** 10.1371/journal.pone.0139991

**Published:** 2015-10-13

**Authors:** Chao Wei, Wenjie He, Li Wei, Chunying Li, Jun Ma

**Affiliations:** 1 State Key Laboratory of Urban Water Resource and Environment, Harbin Institute of Technology, Harbin, Heilongjiang, People's Republic of China; 2 Tianjin Waterworks Group Co., Ltd., Tianjin, People's Republic of China; 3 School of Energy and Civil Engineering, Harbin University of Commerce, Harbin, Heilongjiang, People's Republic of China; NERC Centre for Ecology & Hydrology, UNITED KINGDOM

## Abstract

In this study, high-throughput pyrosequencing was applied on the analysis of the microbial community of activated sludge and biofilm in a lab-scale UV/O_3_- anaerobic/aerobic (A/O) integrated process for the treatment of petrochemical nanofiltration concentrate (NFC) wastewater. NFC is a type of saline wastewater with low biodegradability. From the anaerobic activated sludge (Sample A) and aerobic biofilm (Sample O), 59,748 and 51,231 valid sequence reads were obtained, respectively. The dominant phylotypes related to the metabolism of organic compounds, polycyclic aromatic hydrocarbon (PAH) biodegradation, assimilation of carbon from benzene, and the biodegradation of nitrogenous organic compounds were detected as genus *Clostridium*, genera *Pseudomonas* and *Stenotrophomonas*, class *Betaproteobacteria*, and genus *Hyphomicrobium*. Furthermore, the nitrite-oxidising bacteria *Nitrospira*, nitrite-reducing and sulphate-oxidising bacteria (NR-SRB) *Thioalkalivibrio* were also detected. In the last twenty operational days, the total Chemical Oxygen Demand (COD) and Total Organic Carbon (TOC) removal efficiencies on average were 64.93% and 62.06%, respectively. The removal efficiencies of ammonia nitrogen and Total Nitrogen (TN) on average were 90.51% and 75.11% during the entire treatment process.

## Introduction

Membrane technologies, especially pressure-driven membrane filtration techniques (microfiltration, ultrafiltration, nanofiltration, and reverse osmosis), are efficient, cost-competitive and promising separation methods in the industrial production process; therefore, the membrane separation processes are widely used in wastewater treatment and reclamation [1, 2, 3]. The nanofiltration (NF) technique separates a feed stream into a purified permeate fraction and concentrate. The operating pressure of NF is 5–15 bars, and the concentrate to feed volume ratio is 15–30%, while the NF process does not destroy the pollutants but merely concentrates them into smaller volume. Although the advantages of pressure-driven membrane filtration are obvious, the concentrate could be identified as a major disadvantage. The potential of NF in industrial applications remains underdeveloped because of the disadvantages. In NF, the concentrates contain high concentrations of ions and small organic compounds, which require further treatment [4, 5, 6, 7].

In previous researches, chemical and electro related methods were used on the treatment of NFC. The research emphases are the degradation of pollutants and resources recovery. Advanced oxidation processes (AOPs) were also mentioned in some researches, but they were used as assisted methods. The researches of that the AOPs were direct used on the treatment of NFC were deficient [8, 9, 10, 11, 12]. AOPs are characterised by the production of ·OH radicals, which are extraordinarily reactive species with little selectivity [13, 14, 15]. The AOPs used to oxidise the organic pollutants in wastewater with free radicals have been of value over recent years, and their goal was the mineralisation of the contaminants to carbon dioxide, water and inorganics or at a minimum, destruction of the contaminants’ structures to substances with simpler structures [16, 17]. Chemical oxidation for complete mineralisation is generally expensive; thus, one feasible alternative is the application of AOPs as the pre-treatment to convert the toxic substances with low biodegradability into more biodegradable intermediates, which would then be treated in a biological oxidation process at a considerably lower cost [18].

Biological treatment has the advantages of lower treatment costs with no secondary pollution. It provides the benefits of treatment efficiency, resource recovery, energy consumption, and reduced disposal of sludge, among others. Anaerobic bacteria are capable of transforming most of the organic substances present into biogas with low nutrient demands and minimal sludge formation. Biogas, for example, CH_4_ and H_2_, which is produced in operational time, can be used as energy that can further reduce the consumption and operational costs [19]. Aerobic biological processes are commonly used in the treatment of organic wastewaters to achieve a high degree of treatment efficiency [20]. The aerobic post-treatment improves the removal efficiency and stabilises the fluctuations in the quality of the anaerobic effluent [21]. The biofilm process could treat low concentration wastewater and have good toxic- resistance, the production of residual sludge is low. Based on these theories, the UV/O_3_-anaerobic/aerobic integrated process were used in our research.

In A/O stage of the integrated process, the capacities were achieved by microorganism. So it is necessary to analyse the microbial community structure, and it will contribute to increase the ratio of functional bacteria, and maximize the treatment capacity of the integrated process. At present, there is no report on the analysis of microbial community structure in biological process, which was used on the treatment of saline petrochemical NFC.

The traditional analysis of the microbial community, which consists of cultivation approaches, are time-consuming and frequently onlypresent a minority species of the bacterial in a sample [22]. Recently, high-throughput pyrosequencing has shown promise for the capture of the microbial taxa, and this method can generate enormous amounts of DNA reads through a massively parallel sequencing- by- synthesis approach. This technology has been widely used to analyse the microbial community in various environmental samples [23, 24, 25]. However, the analysis of the microbial community of the activated sludge and biofilm in sequential AOPs and A/O NFC treatment processes has not been reported. The objective of this study was to analyse the bacterial communities in the A/O process of a lab-scale sequential UV/O_3_-A/O integrated process by 454-pyrosequencing. It is useful to understand the process mechanism and improve the treatment effect of that the analysis of microbial community. In our research, the organics degradation and nitrogen reduction were also examined to verify the capacities of the bacteria.

## Materials and Methods

### Experimental setup and operation

**Fig 1:**
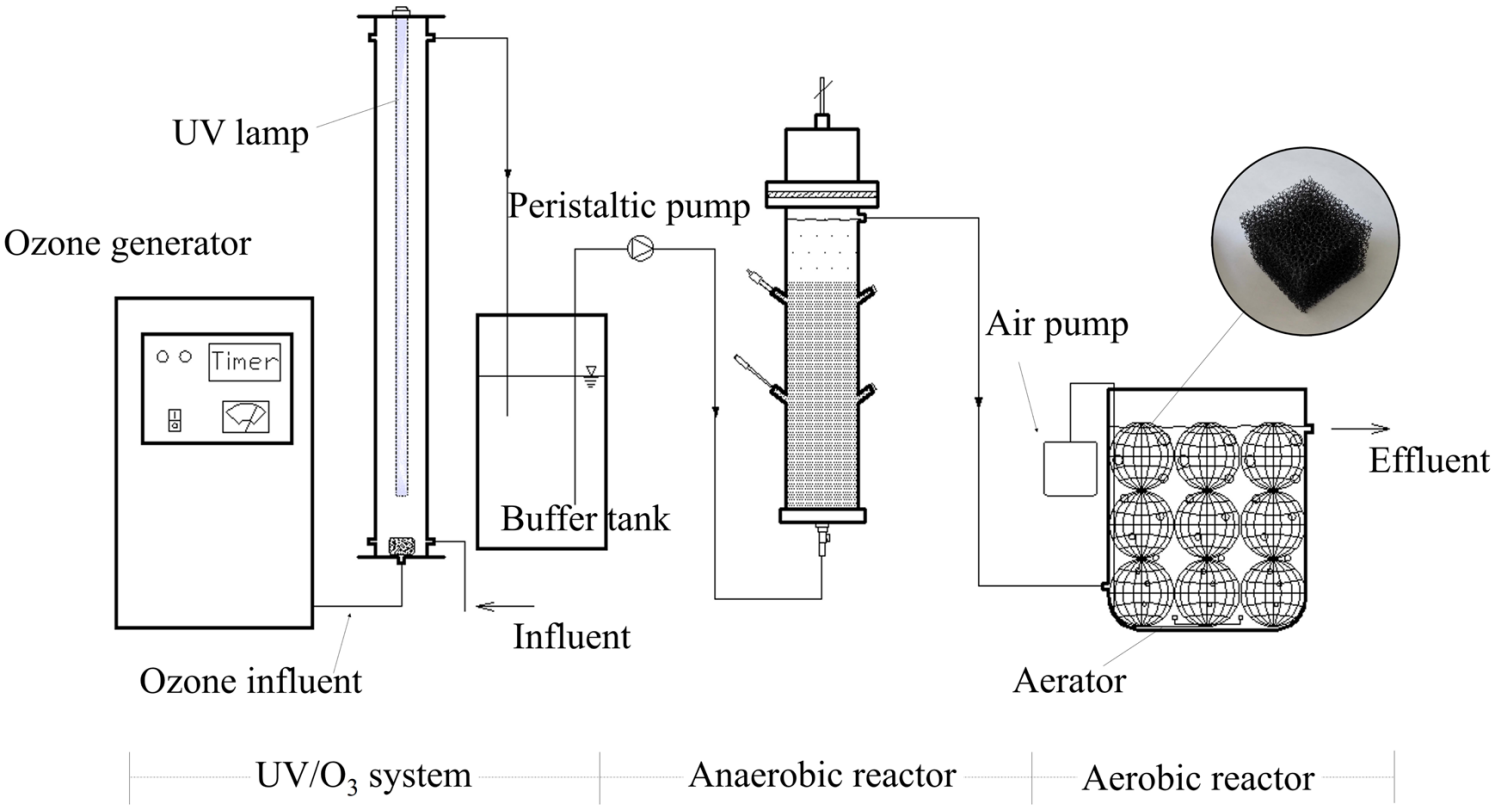
Schematic diagram of the UV/O_3_-A/O integrated process.

A laboratory-scale synthetic glass cylindrical reactor was used in our study. The reactor was 90 cm in height and 9 cm in inner diameter, with a working volume of 4.5 L. The UV lamp (254 nm, 40 W) with quartz shield was set at the centre axis of this reactor. Two openings were connected to a hose and peristaltic pump between the bottom and top of this reactor, which were established as the circulating system (Fig 1). The NFC was pumped into this reactor, and then the NFC was treated using the experimental parameter of 1 g/h O_3_/UV for twenty minutes. The UV/O_3_ system worked under the intermittent operation. After the treatment of UV/O_3_ system, the NFC was stocked in the buffer tank, and the Hydraulic retention time (HRT) of the buffer tank was one day. The treated NFC was pumped into the A/O reactors subsequently. A continuously fed synthetic glass anaerobic internal circulation (IC) and aerobic reactors were used in sequence for our research (Fig 1). The IC reactor had a working volume of 4 L. The aerobic reactor had a working volume of 20 L, and a continuous air supply system was settled at the bottom of this reactor. The aeration rate was controlled at 20: 1 (V _aeration_: V _water_), and the concentration of dissolved oxygen (DO) varied from 3.86 mg/L to 4.73 mg/L in the operational days. Twenty spherical plastic baskets with a 0.1-metre diameter were filled with the degrading bacteria attaching to polyurethane fillers, and they were placed in the aerobic reactor. The anaerobic and aerobic reactors were operated at 37°C using a thermostatic jacket and electric heaters, respectively. HRTs were one and five days in the anaerobic and aerobic reactors, respectively.

The lab-scale A/O system was successfully operated for 60 days. During the start-up period, the A/O system was fed with nutrition containing glucose, NaCl, Na_2_SO_4_, KH_2_PO_4_ and urea. The start-up period lasted approximately 20 days with no NFC addition. After the start-up period, the mixed NFC treated by UV/O_3_ and nutrition (1:1) was fed into the system for 20 days. Then, the treated NFC was fed into this system for further acclimation, and this period continued for 20 days. After acclimation periods, the integrated system operated normally, and this period continued for 60 days. The solid retention time (SRT) of the A/O process was 60 d. The characteristics of the NFC are shown in Table 1. The salinity of the NFC was 6.6‰-7.3‰, and the NFC was a kind of saline petrochemical wastewater that is toxic and has low biodegradability.

**Table 1:** Characteristics of the NFC taken from a petrochemical industry.

	Concentrate	
**COD**	399.10–478.03	mg/L
**TOC**	146.48–166.49	mg/L
**NH**_**4**_^**+**^ **-N**	29.74–38.75	mg/L
**TN**	61.04–86.97	mg/L
**Cl** ^**-**^	418.44–559.52	mg/L
**SO**_**4**_^**2-**^	851.16–947.04	mg/L
**pH**	8.27–8.41	
**Salinity**	6.6‰–7.3‰	

### Analysis methods

The COD samples in all of the stage were detected using the potassium dichromate titrimetric method according to the Standard Methods for the Examination of Water and Wastewater [26]. TOC and TN were detected using a TOC/ TN analyser (Shimadzu TOC-5000A). Ammonia nitrogen were analysed by using an ion chromatograph (HIC-20A super) according to standard method. DO was analysed by using portable DO analyzer (HACH, HQ30d).

Gas chromatography (GC, Agilent Technologies 7890A) coupled with mass spectrometry (MS, Agilent Technologies 7890A) were used to detect the decreasing progress of the organics. The buffer gas was highly pure nitrogen, and diluted samples were prepared using methyl tert-butyl ether (MTBE). The operational conditions of GC-MS were the following: the injector temperature was 250°C, and the column initial temperature was maintained at 35°C for 3 min. Then, the temperature was gradually increased to 280°C at a rate of 10°C/min and held for 5 min, and the ion source temperature of MS was 240°C.

### DNA extraction, PCR and pyrosequencing

The sludge samples of Day 60 were collected in the anaerobic and aerobic reactors, respectively. We collected the activated sludge in anaerobic reactor, and marked it as Sample A. Several blocks of fillers were taken out from the aerobic reactor, and they were shaken in deionized water. We collected the suspend solid in deionized water, and marked it as Sample O.

The DNA was extracted using the PowerSoil DNA extraction kit (MO BIO Laboratories, Inc., Carlsbad, CA) according to the instruction, and the DNA was amplified using universal bacterial primer 8F (5’-3’ AGAGTTTGATCCTGGCTCAG) and 533R (5’-3’ TTACCGCGGCTGCTGGCAC) covering the V1 and V3 regions. Different ten-nucleotide barcode sequences and pyrosequencing adapters were added at the 5’ end of the universal bacterial primer. The PCR products were purified using the TaKaRa Agarose Gel DNA Purification Kit (TaKaRa, China) and quantified using NanoDrop. 454 pyrosequencing was carried out using the Roche 454 FLX Titanium platform at the National Human Genome Centre of China at Shanghai, China (CHGC).

### Sequence analysis

The sequences were filtered for quality and length. Initially, the base mismatches of sequencing primers were examined, and the sequences which they were no more than 2 bp, were reserved. Then, the average base quality was examined, and when the average base quality in any continuous 50 bp read was less than 20 (error rate greater than 1%), the 50 bp read and the followed bases were removed. The containing ambiguous “N” and the followed bases were removed. Finally, the sequences shorter than 200 bp in length and containing repeat bases more than 10 bp were removed, and the chimeras generated in PCR amplification were filtered out to form high quality sequences. The high quality sequences were assigned to samples according to barcodes. The sequences were aligned using Mothur ver. 1.17.0 and clustered into operational taxonomic units (OTUs) at 90, 95 and 97% similarities. The OTUs (at 97% similarity) of the samples were used for coverage, Shannon (diversity), Chao (richness), ACE, Simpson and rarefaction curve analysis. Taxonomic classification of the sequences were performed using the RDP Classifier of the Ribosomal Database Project (RDP), the National Centre for Biotechnology Information (NCBI) BLAST, and the Greengenes databases at 70% confidence threshold. The sequence data have been submitted to NCBI Sequence Read Archive database (Accession Numbers: SRR2420282 and SRR2420283 for Sample A and Sample O, respectively).

## Results

### Performance of the UV/O_3_-A/O integrated process

**Fig 2:**
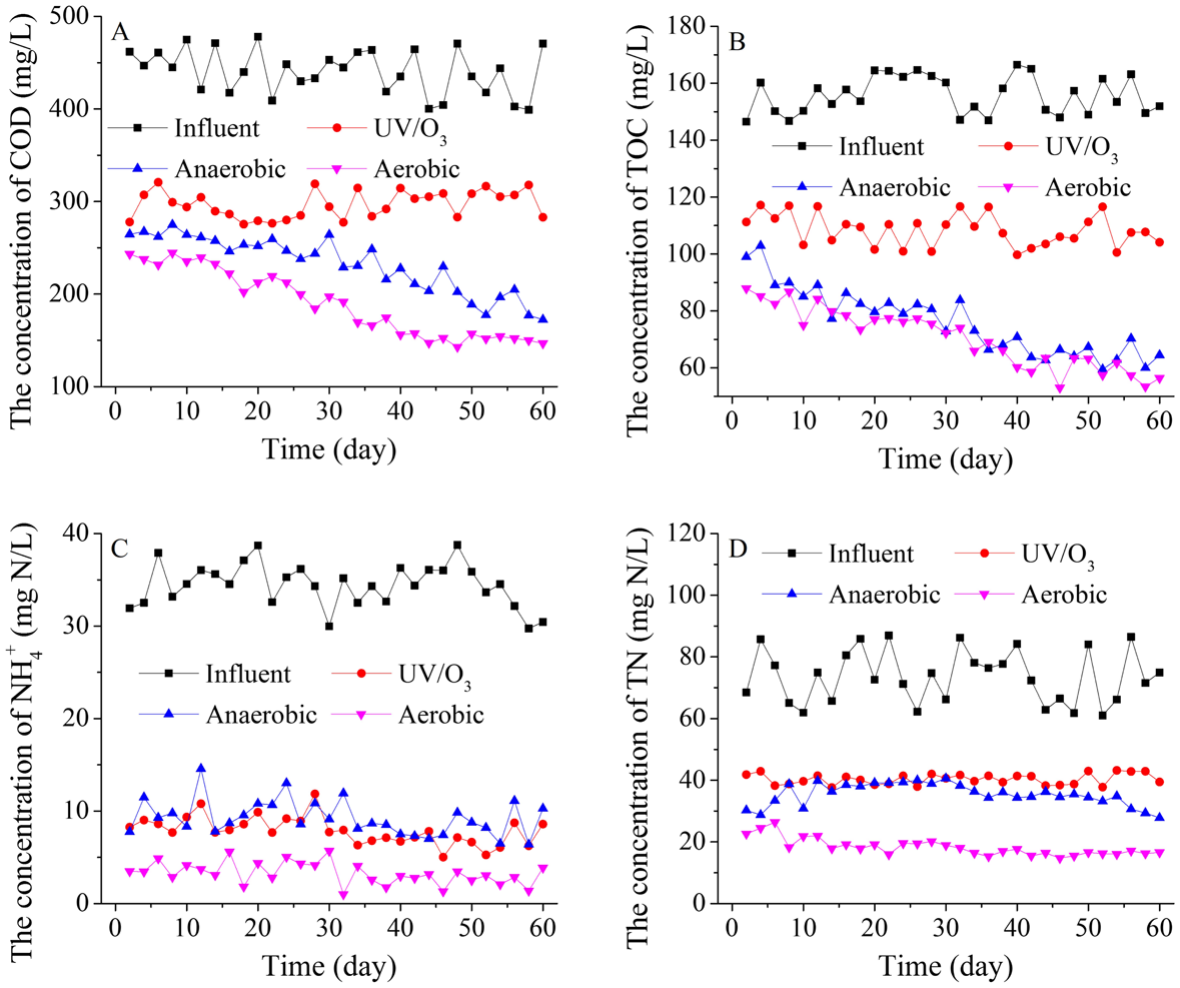
The performance of the integrated process. COD, TOC, ammonia nitrogen and TN removal efficiencies of the integrated process were shown in A), B), C) and D), respectively.

During the 60-day treatment period, the COD, TOC, TN and ammonia nitrogen concentrations decreased and stabilised during the last treatment stage. At the last treatment stage, the COD removal efficiencies ranged from 62.25% to 69.65%, while the COD removal efficiencies were low for the aerobic stage. At the last twenty operational days, the COD concentration of effluent on average was 151.15 mg/L, and the total removal efficiency on average was 64.93%, while the COD removal efficiencies in the anaerobic stage and aerobic stage on average were 24.95% and 10.49%, respectively (Fig 2A). During the 60 operational days, the variation trend of TOC was the same as it of COD. The TOC concentration of effluent decreased and gradually stabilized. The total TOC removal efficiency on average was 62.06%, while the TOC concentration of effluent on average was 58.80 mg/L at the last twenty operational days (Fig 2B).

The ammonia nitrogen and TN concentrations decreased obviously during the treatment of UV/O_3_. On average, the ammonia nitrogen and TN concentrations decreased from 34.43 mg/L to 7.89 mg/L and from 73.66 mg/L to 40.35 mg/L, respectively. Although there were no obvious changes of ammonia nitrogen and TN concentrations in anaerobic stage, they could be removed in aerobic reactor. On average, the ammonia nitrogen and TN concentrations of effluent were 3.27 mg/L and 18.28 mg/L, and the total removal efficiencies of them in the integrated process were 90.51% and 75.11%, respectively (Fig 2C and 2D).

### The analysis of the organic pollutants by GC-MS

**Fig 3:**
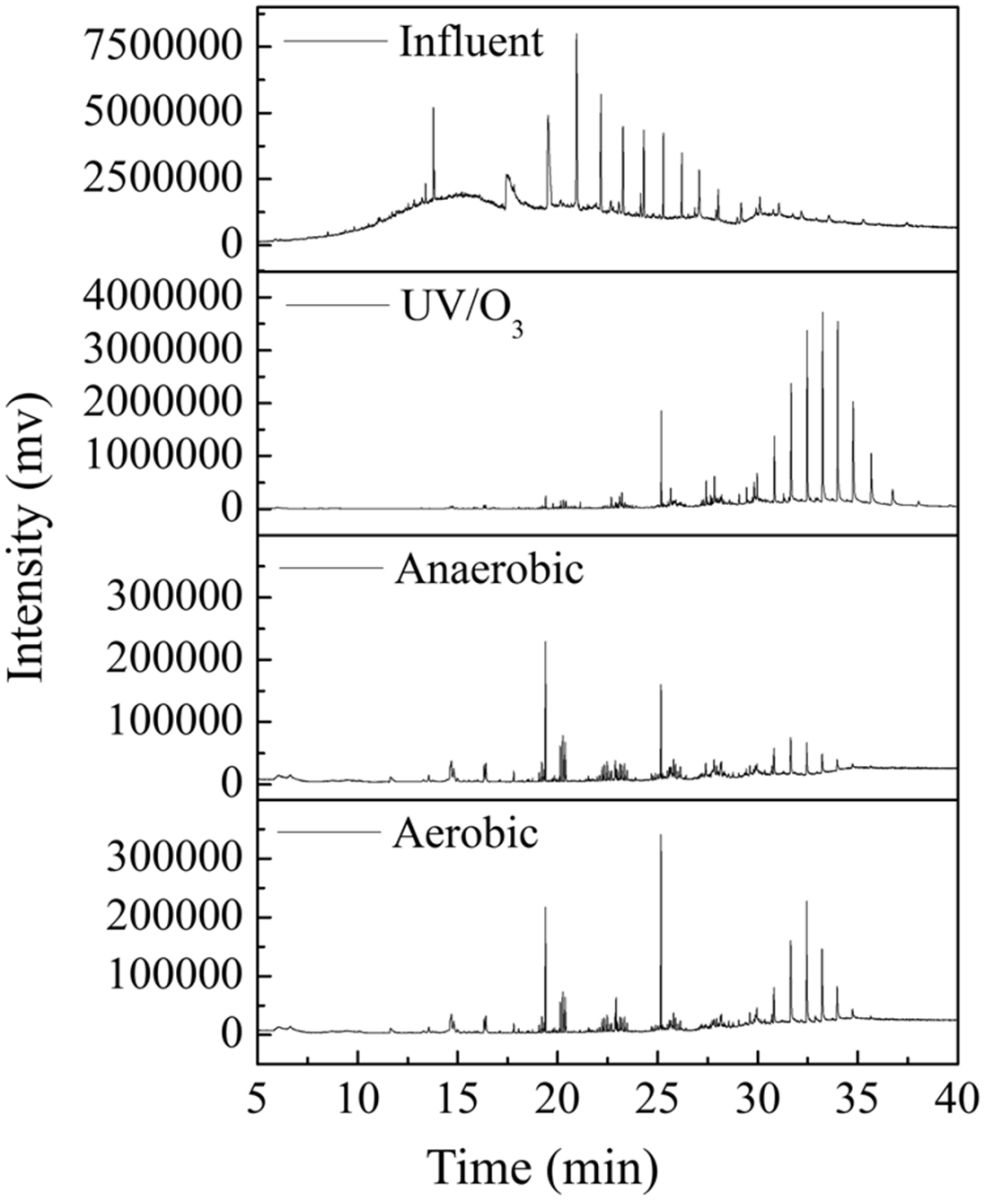
The gas chromatograms of every treatment process effluents.

We analysed the organic pollutants by GC-MS throughout this treatment process, and the gas chromatograms are shown in Fig 3. In the treatment process, the component of organic pollutants changed, and the contrast of the primary organic pollutants is shown in S1 Table. Some organics, for instance Pyrene, hexadecahydro-, Butylated Hydroxytoluene, 1-benzylindole, Benzo[e]pyrene, 4-Nitro-4'-chlorodiphenylsulfoxide, Cyclodecasiloxane homologen were detected. These organics contain polycyclic aromatic or other cyclic hydrocarbon, and the structures of the organic pollutants in this NFC are complex and have a low degradability. After the treatment of UV/O_3_, these organics disappeared, so it could destroy the complex structures of organics in the NFC. The component of organic pollutants further changed after the treatment of A/O process.

### Microbial diversity

**Fig 4:**
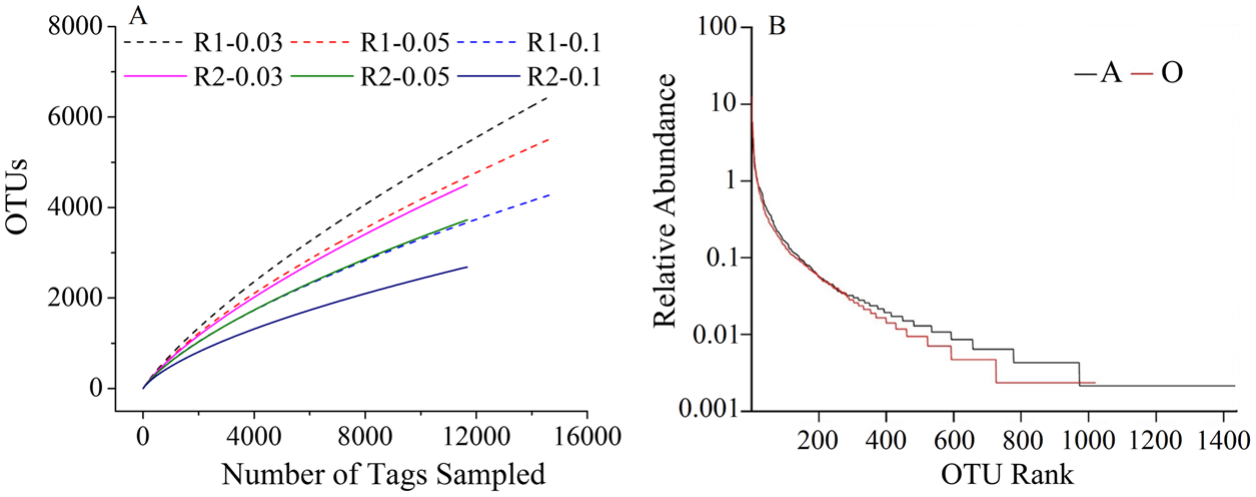
Rarefaction analysis of the different samples. a) Rarefaction curves are depicted at 3%, 5% and 10% dissimilarity level; b) Rank-abundances show pyrosequencing abundances of different samples.

The rarefaction analysis of the bacterial communities derived from the anaerobic (Sample A) and aerobic (Sample O) samples is depicted as having a 3%, 5% and 10% dissimilarity. At 10% genetic distance, the two curves approached saturation, indicating that the sequencing nearly covered the OTUs in the two samples (Fig 4A). The coverage index of the two samples approached 99%, which indicated that the recovered sequences well represent the microbial diversity in the two samples. The well-distributed rank-abundance curves showed that the distribution of OTUs derived from Sample A was wider than that from Sample O, which indicate that the microbial diversity of Sample A was higher than that of Sample O (Fig 4B). In addition, the values of the ACE, Chao and Shannon indices further supported this result (S2 Table).

The unique and shared OTUs were represented by a Venn diagram, and the results showed that 304 OTUs were common for the two samples, and 1,135 and 716 OTUs were unique to Sample A and Sample O, respectively (S1 Fig). The NFC was pumped into the continuously fed reactors in our experiment; thus, the microorganisms in the anaerobic reactor could transfer to the aerobic reactor and affect its microbial diversity.

### Microbial community

**Fig 5:**
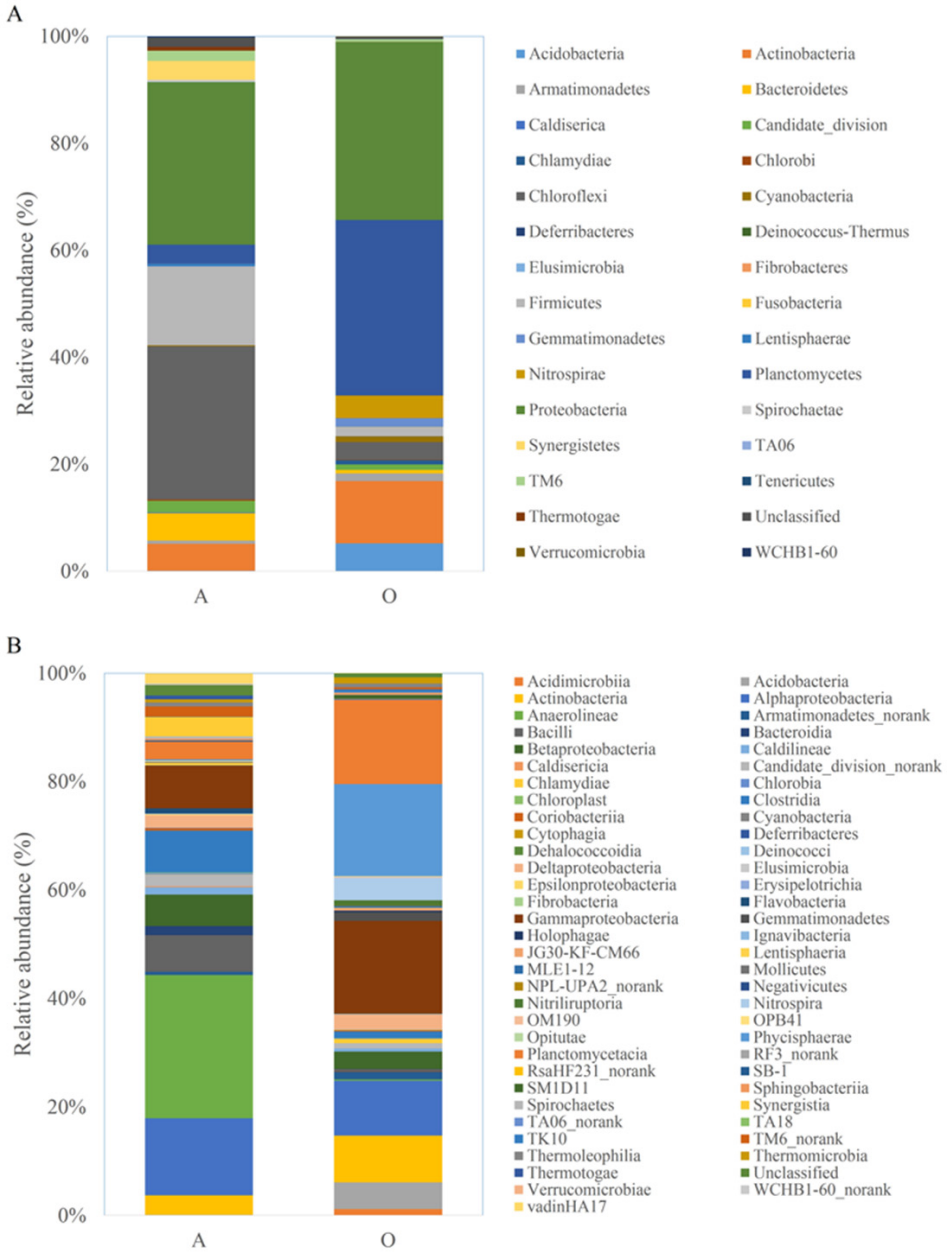
Microbial composition at the phylum and class level. Color-coded bar plot showing the microbial phylum relative abundance across Sample A and Sample O.

At the phylum level, the microbial communities and the dominant phyla of Sample A and Sample O were different (Fig 5A). The microbial communities in the two samples were primarily related to the environmental parameter of DO, and the difference of DO was caused by the anaerobic and aerobic conditions. *Proteobacteria* was the most dominant phylum in the two samples, accounting for 30.40% and 33.20% in Sample A and Sample O, respectively. In Sample A, the other dominant phyla were *Chloroflexi* (28.62%) and *Firmicutes* (14.75%), and these three groups were dominant (73.77%) in the bacterial communities of Sample A, followed by a few other major phyla (average abundance > 1%), including *Bacteroidetes* (5.08%), *Actinobacteria* (5.03%), *Planctomycetes* (3.56%), *Synergistetes* (3.52%), and *TM6* (1.90%). In Sample O, the other dominant phyla were *Planctomycetes* (32.83%) and *Actinobacteria* (11.66%), and these three groups were dominant (77.69%) in the bacterial communities of Sample O, followed by a few other major phyla (average abundance > 1%), including *Acidobacteria* (5.20%), *Nitrospirae* (4.18%), *Chloroflexi* (3.27%), *Firmicutes* (1.71%), *Gemmatimonadetes* (1.63%), *Armatimonadetes* (1.43%). The abundances of the other phyla were < 1% in the two samples (S3 Table).

At the class level, the microbial communities of Sample A and Sample O were different (Fig 5B). In Sample A, *Anaerolineae* (24.60%) within phylum *Chloroflexi* was the most dominant class. Within phylum *Proteobacteria*, *Alphaproteobacteria* (14.22%) was the most dominant group, followed by the *Gamma-* (7.86%), *Beta-* (5.78%), and *Delta-* (3.23%) subdivisions. This finding was different from the results of Sample O, which showed that *Gammaproteobacteria* (17.09%) was the dominant group, followed by the *Alpha-* (10.12%), *Beta-* (3.23%), and *Delta-* (2.78%) subdivisions. In addition to the four classes of the *Proteobacteria*, the two samples shared other classes, including *Clostridia* and *Actinobacteria*. In Sample A, the other dominant classes were *Bacilli* (6.74%), *Synergistia* (3.51%), *vadinHA17* (1.91%), *TM6_norank* (1.89%), *Bacteroidia* (1.70%), and *Caldilineae* (1.36%). In Sample O, classes *Phycisphaerae* (16.96%) and *Planctomycetacia* (15.59%) within phylum *Chloroflexi* were dominant. The other dominant classes were *Acidobacteria* (4.95%), *Nitrospira* (4.17%), *Gemmatimonadetes* (1.63%), *Armatimonadetes_norank* (1.43%), *Thermomicrobia* (1.43%), and *Acidimicrobiia* (1.16%) in Sample O (S4 Table).

**Fig 6:**
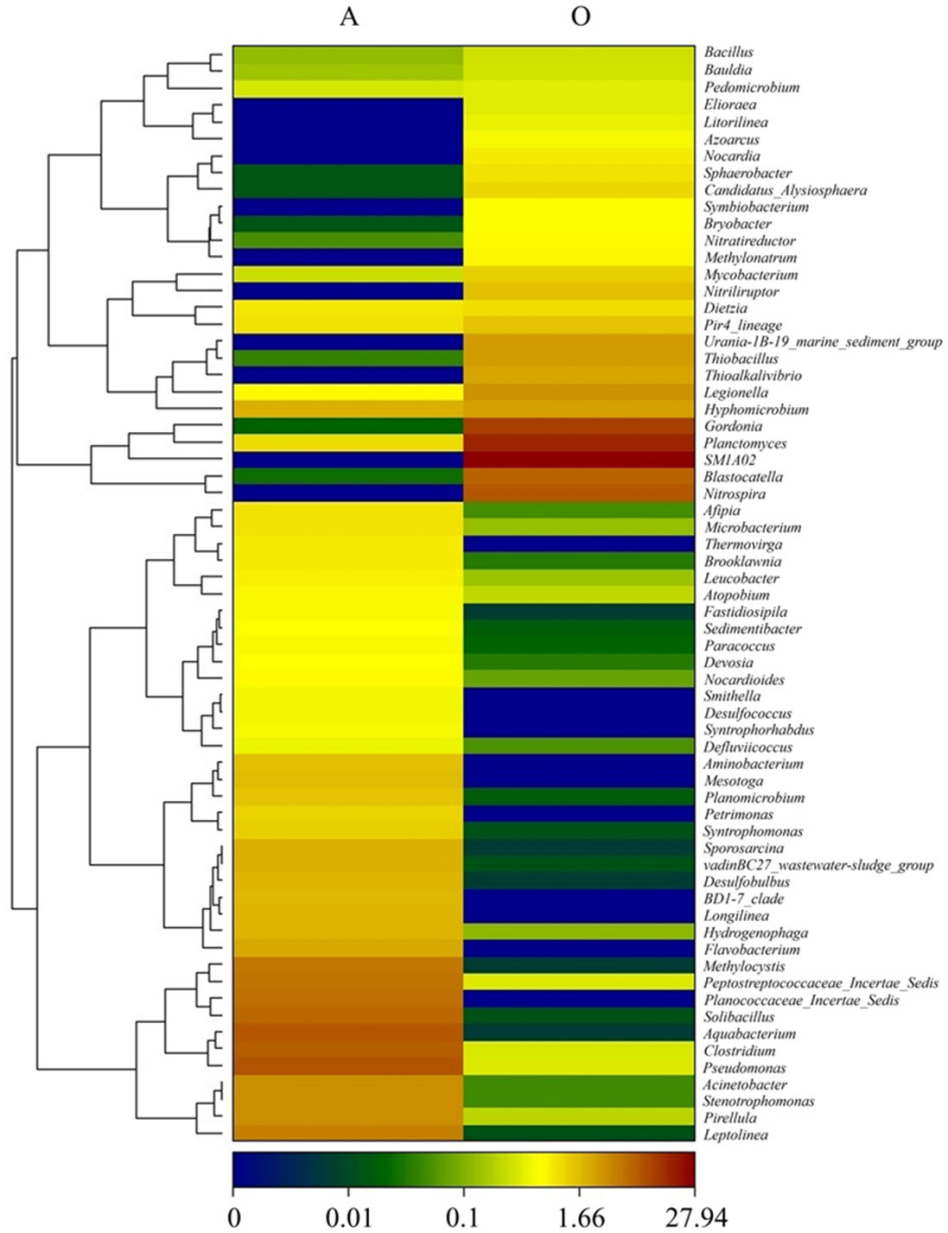
Relative abundance of genera in Sample A and Sample O. The heatmap color-coded bar plot depicts the relative abundance of each sample. The relative abundance for microbial genera are indicated by color intensity from low (blue) to high (red) with the legend indicated at the bottom.

The hierarchical heatmap is based on the dominant genera (abundance > 1‰) in each sample. Although the genera of Sample A and Sample O were different, the two samples shared certain genera (Fig 6). The most dominant genus in Sample A was *Pseudomonas* (3.24%), while *SM1A02* (15.32%) was the most dominant genus in Sample O. The two samples shared certain dominant genera, including *Candidate_division_BRC1_norank*, *Planctomyces*, *Hyphomicrobium*, and *Armatimonadetes_norank*, but their abundances were different in the two samples. The other dominant genera in Sample A were *Aquabacterium* (2.99%), *Clostridium* (2.98%), *Solibacillus* (2.47%), *Planococcaceae_Incertae_Sedis* (2.24%), *Peptostreptococcaceae_Incertae_Sedis* (2.06%), *Methylocystis* (1.96%), *vadinHA17_norank* (1.91%), *TM6_norank* (1.89%), *Leptolinea* (1.74%), *Pirellula* (1.44%), *Stenotrophomonas* (1.42%), *Acinetobacter* (1.41%), and *Longilinea* (1.31%). The other dominant genera in Sample O were *KCM-B-112_norank* (5.98%), *Gordonia* (5.89%), *Nitrospira* (4.17%), *Blastocatella* (3.58%), *Legionella* (1.80%), *Thiobacillus* (1.557%), *Thioalkalivibrio* (1.37%), *MSB-1E8_norank* (1.155%), *Urania-1B-19_ marine_ sediment_group* (1.50%), and *GR-WP33-30_norank* (1.15%). Meanwhile, the *uncultured*, *unclassified* and *uncultured_norank* accounted for 7.20%, 6.89%, 5.14%, respectively. These groups account for a large proportion, which most likely play a significant yet unknown or less understood role (S5 Table). The abundances of the species are shown in S6 Table.

## Discussion

The petrochemical NFC used in our research is toxic and has low biodegradability; thus, the conventional biological treatments do not consistently obtain satisfactory results. Chemical oxidation for complete mineralisation is generally expensive; therefore, we applied the UV/O_3_ as a pre-treatment to convert the toxic substances with low biodegradability into more biodegradable intermediates, which were then treated in an A/O biological oxidation process for a considerably reduced cost. The NFC was a type of saline petrochemical wastewater containing PAHs and other organic contamination with benzene.

It is important to establish the relationship between the microbial community structure and the bioreactor performance. In our research, we used 454-pyrosequencing to analyse the overall microbial community in this sequential anaerobic/aerobic process. *Proteobacteria* (*Alpha-*, *Gamma-*, *Beta-*, and *Delta-* subdivision, arranged by abundance) was the most dominant phylum in Sample A, and *Proteobacteria* (*Gamma-*, *Alpha-*, *Beta-*, and *Delta* subdivisions, arranged by abundance) was the most dominant phylum in Sample O. The phylum accounted for 30.40% and 33.20% of the total effective bacterial sequences in the two samples, respectively. In addition, the two samples shared certain common phyla, but the abundances of these phyla were different. In Sample A, the dominant phyla were *Chloroflexi*, *Firmicutes*, *Actinobacteria* and *Planctomycetes*, accounting for 28.62%, 14.75%, 5.03% and 3.56%, respectively. Correspondingly, the four phyla accounted for 3.27%, 1.71%, 11.66% and 32.83% in Sample O. In Sample A, *Proteobacteria*, *Chloroflexi*, and *Firmicutes* were the three dominant phyla, with their sum accounting for 73.77%. In Sample O, *Proteobacteria*, *Chloroflexi*, and *Firmicutes* were the three dominant phyla, with their sum accounting for 77.69%. The obvious differences in the bacterial community were caused by different operational conditions; thus, the effects of the reactors were different.

The phyla *Bacteriodetes* (5.08%), *Actinobacteria* (5.03%), *Synergistes* (3.52%) and *Firmicutes* (14.75%) detected in our study have wide ecological niches in both natural and industrial environments. Most of the close relatives of the OTUs are chemoorganotrophic, and some of them exist in contaminated environments containing complex organic matters [22]. The organic in the NFC could provide substances for the mechanism of these microorganism, so these phyla may play roles in the degradation of the organic pollutants in the NFC. *Leptolinea* and *Longilinea* were detected in Sample A. The two genera are members of the *Anaerolineae* class, accounting for 24.60% at the class level, which was a dominant class in Sample A. The class *Anaerolineae* is a member of the *Chloroflexi* phylum in Sample A; the phylum accounted for 28.62% at the phylum level. The *Anaerolineae* class shares common physiological and morphological traits, such as anaerobic growth on carbohydrates; thus, the two genera were related to the anaerobic condition [27, 28]. The *Clostridium* genus accounted for 2.98% in Sample A, and it is a member of the *Clostridia* class. The genus was detected in Sample A and Sample O, which accounted for 7.75% and 1.25%, respectively. Wang et al. have revealed that *Clostridia* was a class primarily composed of chemoorganotrophic species metabolising carbohydrates, alcohols, amino acids, and other organic compounds [22]. The genus was detected in our reactors, which would be related to the biodegradation of the organic pollutants. It is difficult to biodegrade the PAHs contained in this NFC. Lu has reported that several aerobic pure cultures degrading naphthalene, phenanthrene, or pyrene as the sole carbon source have been isolated, most of them belonging to *Pseudomonas*, *Alcaligenes*, *Mycobacterium*, *Rhodococcus*, *Neptunomonas*, *Stenotrophomonas*, *Sphingomonas*, *Cycloclasticus*, *plus Aeromonas*, *Corynebacterium* and *Micrococcus* [29]. Many of the bacteria capable of degrading oil are Gram-negative, and certain alkane- and aromatic-degrading bacteria were classified into Gram-negative *Pseudomonas* [24]. The genera *Pseudomonas* and *Stenotrophomonas* were detected in Sample A, and *Pseudomonas* was the most dominant genus in the anaerobic reactor, accounting for 3.24%. The *Stenotrophomonas* genus was also a dominant genus in Sample A, accounting for 3.24%. The existence of genera *Pseudomonas* and *Stenotrophomonas* would enable the anaerobic process to degrade the PAHsThe biodegradation of alkanes was found to correlate with groups related to denitrification and the sulphate reduction [24, 29]. In addition, the *Acinetobacter* from *Gammaproteobacteria* contributes to the mineralisation of aromatic compounds [24]. Phylotypes related to the *Zoogloea*, *Ferribacterium*, *Aquabacterium* and *Hydrogenophaga* genera within the *Betaproteobacteria* class predominantly assimilated carbon from benzene [30]. In Sample A, *Aquabacterium* was the dominant genus, and the abundance accounted for 2.99%. The genus *Hydrogenophaga* accounted for 0.81%, and the *Betaproteobacteria* class accounted for 5.78% at the class level. The low- degradability organics contained in the influent of the A/O process were mainly long-chain alkane and polycyclic aromatic (S1 Table). The alkane and aromatic- degrading microorganism were detected, and they could transfer these hydrocarbons into organics with simpler structures. These organics could be served as substances for the metabolism of the microorganism, which metabolized wide range of substances, such as genus *Clostridium*. Through these microorganism, the alkane and aromatic could be degrade, and the process accomplished the removal of COD and TOC.

The nitrite-oxidising bacterium genus *Nitrospira* (accounting for 4.17%) was detected in Sample O. *Nitrospira* is the most important nitrite-oxidising bacteria (NOB), which is adapted to live under significant substrate limitation [31, 32]. The genus *Hyphomicrobium* (accounting for 1.43%) was detected in Sample O, and it was related to nitrogen metabolism. The presence of genera *Bradyrhizobium*, *Hyphomicrobium*, *Microcystis* and *Sphingobium* might play important roles in the biodegradation of nitrogenous organic compounds in waters [33]. In Sample O, the sulphide-oxidising related genus *Thioalkalivibrio* (accounting for 1.37%) was detected, and it is a type of nitrate-reducing, sulphide-oxidising bacteria (NR-SOB). Under limited-oxygen conditions, sulphate, nitrate and organic carbon can be successfully removed simultaneously by NR-SOB [34]. These genera *Nitrospira*, *Hyphomicrobium*, *Thioalkalivibrio* contributed to the nitrogen transformation in aerobic reactor, which the genera *Hyphomicrobium*, *Thioalkalivibrio* could realize the function of denitrification. In this research, we chose biofilm process as the aerobic treatment. Although we provided aeration to maintain the appropriate oxygen condition in the aerobic reactor, the limited oxygen area still existed within the fillers. The biofilm attaching to fillers could be divided into different layers according to the DO level, and the inner fillers in the plastic may stay at anoxic condition. So the biofilm process could provide conditions for the existence of these bacteria. The denitrification and nitrification bacteria existed simultaneously, and the TN was removed in the aerobic reactor. So we speculated that simultaneous nitrification and denitrification (SND) occurred in the aerobic reactor. The appropriate DO condition for the living of *Thioalkalivibrio* is contradictory to the DO level in the aerobic reactor. As a result, the genus *Thioalkalivibrio* could only live at limited area. The abundance of denitrification bacteria were not very high, so the SND was not the primary pathway of nitrogen transformation in the aerobic reactor.

## Conclusion

High-throughput 454-pyrosequencing provides sufficient sequencing for the analysis of the microbial community. The PAH- and benzene-degrading bacteria were detected, and the nitrogenous organic compounds-degrading, sulphate-reducing, nitrate-oxidising bacteria also existed in this A/O process. The performances of these reactors were consistent with the microbial community, and this integrated process is effective for petrochemical NFC treatment. The microbial diversities’ analysis is helpful to understand the mechanisms of organics degradation and nitrogen reduction in the integrated system.

## Supporting Information

S1 Fig Venn diagram show unique and shared OTUs between different samples. (TIF)

S1 Table Main pollutants contrast during the treatment of NFC. (DOC)

S2 Table Diversity indexes of the two samples. (DOC)

S3 Table The abundances of phylum (bacterial count > 200) in the two samples. Arranged according to the abundance.
(DOC)

S4 Table The abundances of classes (bacterial count > 200) in the two samples. Arranged according to the abundance.
(DOC)

S5 Table The abundances of genera (bacterial count > 200) in the two samples. Arranged according to the abundance.
(DOC)

S6 Table The abundances of species (bacterial count > 200) in the two samples. Arranged according to the abundance.
(DOC)
