# Supplementary figures and images for: The Analysis of a Microbial Community in the UV/O3-Anaerobic/Aerobic Integrated Process for Petrochemical Nanofiltration Concentrate (NFC) Treatment by 454-Pyrosequencing
Source: PLoS One. 2015 Oct 13;10(10):e0139991. doi: 10.1371/journal.pone.0139991 (PMC4603877; doi:10.1371/journal.pone.0139991)

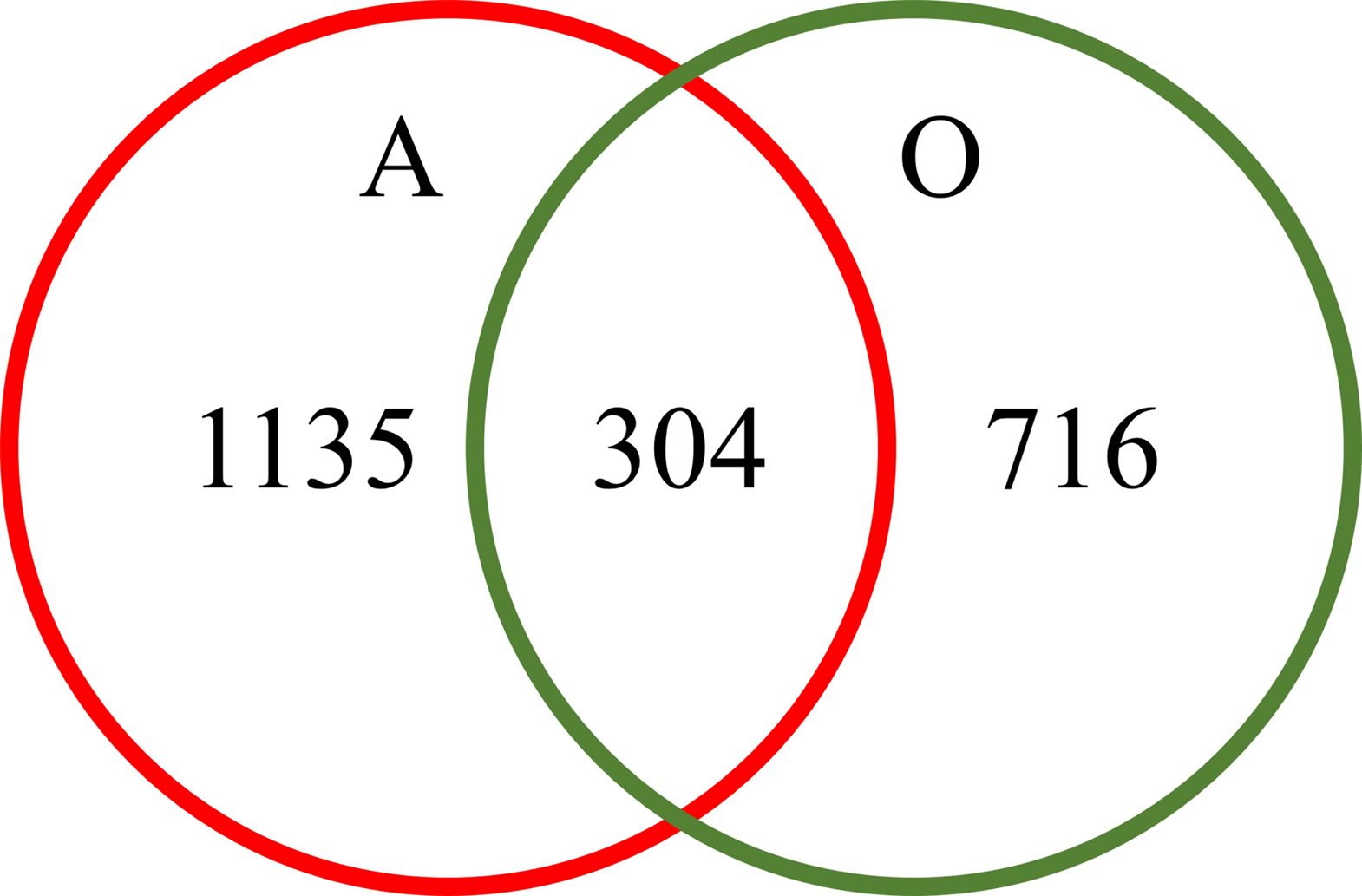

Supplement: S1 Fig — (TIF) [file pone.0139991.s001.tif]
