# Supplementary material for: The Analysis of a Microbial Community in the UV/O3-Anaerobic/Aerobic Integrated Process for Petrochemical Nanofiltration Concentrate (NFC) Treatment by 454-Pyrosequencing
Source: PLoS One. 2015 Oct 13;10(10):e0139991. doi: 10.1371/journal.pone.0139991 (PMC4603877; doi:10.1371/journal.pone.0139991)
Supplement: S1 Table — (DOC) [file pone.0139991.s002.doc]

Supporting Information

S1 Table Main pollutants contrast during the treatment of NFC.

| **RT (min)** | **Name** | **Mol. Weight** | **Influent** | **UV/O_3_** | **Anaerobic** | **Aerobic** |
| --- | --- | --- | --- | --- | --- | --- |
| 3.116 | Silanediol, dimethyl- | 92.029 |  |  | + | + |
| 13.398 | Pyrene, hexadecahydro- | 218.203 | + |  |  |  |
| 13.792 | Butylated Hydroxytoluene | 220.183 | + |  |  |  |
| 14.686 | Cyclotetrasiloxane, octamethyl- | 296.075 |  |  | + |  |
| 16.326 | Cyclohexane, 3-ethyl-5-methyl-1-propyl- | 168.188 |  |  | + |  |
| 16.413 | Trichloroacetic acid, 2-ethylhexyl ester | 274.029 |  |  | + |  |
| 17.459 | Cyclodecasiloxane, eicosamethyl- | 740.188 | + |  |  |  |
| 19.214 | Octadecane, 2,6-dimethyl- | 282.329 |  |  | + |  |
| 19.393 | Benzene, 1,3-bis(1,1-dimethylethyl)- | 190.172 |  | + | + | + |
| 19.523 | Cyclooctasiloxane, hexadecamethyl- | 592.15 | + |  |  |  |
| 20.133 | Cyclohexane, 1,2,4-trimethyl- | 126.141 |  |  | + | + |
| 20.26 | 4-Isopropyl-1,3-cyclohexanedione | 154.099 |  |  | + | + |
| 20.271 | 1-Ethyl-2,2,6-trimethylcyclohexane | 154.172 |  | + |  |  |
| 20.375 | Cyclohexane, 1,2-diethyl-1-methyl- | 154.172 |  |  | + | + |
| 20.954 | Silane,[[4-[1,2-bis[(trimethylsilyl)oxy]ethyl]-1,2-phenylene]bis(oxy)]bis[trimethyl- | 458.216 | + |  |  |  |
| 22.163 | Cyclononasiloxane, octadecamethyl- | 666.169 | + |  |  |  |
| 22.466 | Tridecane, 3-methyl- | 198.235 |  |  | + |  |
| 22.674 | Eicosane | 282.329 |  | + |  |  |
| 22.877 | Phenol, 2,4-bis(1,1-dimethylethyl)- | 206.167 |  |  | + |  |
| 22.929 | Butylated Hydroxytoluene | 220.183 |  |  |  | + |
| 23.059 | 1-benzylindole | 207.105 | + |  |  |  |
| 23.09 | Benzoic acid, 4-ethoxy-, ethyl ester | 194.094 |  | + |  |  |
| 23.223 | Heptadecane, 2-methyl- | 254.297 |  | + |  |  |
| 23.264 | Cyclononasiloxane, octadecamethyl- | 666.169 | + |  |  |  |
| 23.344 | Octadecane, 1-(ethenyloxy)- | 296.308 |  |  | + |  |
| 23.356 | Sulfurous acid, 2-propyl tridecyl ester | 306.223 |  | + |  |  |
| 24.144 | Benzo[e]pyrene | 252.094 | + |  |  |  |
| 24.309 | 4-Nitro-4'-chlorodiphenylsulfoxide | 280.991 | + |  |  |  |
| 25.164 | O-Butyl,O-1,2,2-trimethylpropyl methylphosphonate | 236.154 |  |  | + | + |
| 25.181 | Propane, 1,1,2,2-tetrachloro- | 179.907 |  | + |  |  |
| 25.279 | Cyclodecasiloxane, eicosamethyl- | 740.188 | + |  |  |  |
| 25.649 | Octadecane, 2-methyl- | 268.313 |  | + |  |  |
| 25.794 | Eicosyl heptafluorobutyrate | 494.299 |  |  | + |  |
| 26.208 | 4-Nitro-4'-chlorodiphenylsulfoxide | 280.991 | + |  |  |  |
| 26.857 | 1,1':3',1''-Terphenyl, 5'-phenyl- | 306.141 | + |  |  |  |
| 27.038 | Methanol, [4-(1,1-dimethylethyl)phenoxy]-, acetate | 222.126 | + |  |  |  |
| 27.423 | Octadecane, 1-iodo- | 380.194 |  | + |  |  |
| 27.642 | 2-Furanacetic acid, .alpha.-hydroxy- | 142.027 |  | + |  |  |
| 27.833 | Octacosane | 394.454 |  | + |  |  |
| 28.025 | 4-Nitro-4'-chlorodiphenylsulfoxide | 280.991 | + |  |  |  |
| 28.133 | Eicosane | 282.329 |  | + |  |  |
| 28.197 | Hexadecane, 2-methyl- | 240.282 |  | + |  |  |
| 29.176 | Cyclooctasiloxane, hexadecamethyl- | 592.15 | + |  |  |  |
| 29.45 | Tetratriacontane | 478.548 |  | + |  |  |
| 29.82 | Heneicosane | 296.344 |  | + |  |  |
| 30.105 | Octasiloxane,1,1,3,3,5,5,7,7,9,9,11,11,13,13,15,15-hexadecamethyl- | 578.171 | + |  |  |  |
| 30.819 | Heneicosane | 296.344 |  | + | + | + |
| 31.059 | Octasiloxane,1,1,3,3,5,5,7,7,9,9,11,11,13,13,15,15-hexadecamethyl- | 578.171 | + |  |  |  |
| 31.651 | Eicosane | 282.329 |  |  | + | + |
| 33.996 | Heptadecane | 240.282 |  | + |  |  |

“+” presented that the substance was detected.
