# Supplementary material for: The Analysis of a Microbial Community in the UV/O3-Anaerobic/Aerobic Integrated Process for Petrochemical Nanofiltration Concentrate (NFC) Treatment by 454-Pyrosequencing
Source: PLoS One. 2015 Oct 13;10(10):e0139991. doi: 10.1371/journal.pone.0139991 (PMC4603877; doi:10.1371/journal.pone.0139991)
Supplement: S2 Table — (DOC) [file pone.0139991.s003.doc]

Supporting Information

S2 Table Diversity indexes of the two samples.

| **Sample** | **Valid** | **Trimed** | **Coverage index** | **ace** | **chao** | **shannon** | **simpson** |
| --- | --- | --- | --- | --- | --- | --- | --- |
| **A** | 59748 | 48061 | 0.99 | 1825.55 | 1851.11 | 5.05 | 0.02 |
| **O** | 51231 | 42338 | 0.99 | 1322.90 | 1321.32 | 4.36 | 0.02 |
