# Supplementary material for: The Analysis of a Microbial Community in the UV/O3-Anaerobic/Aerobic Integrated Process for Petrochemical Nanofiltration Concentrate (NFC) Treatment by 454-Pyrosequencing
Source: PLoS One. 2015 Oct 13;10(10):e0139991. doi: 10.1371/journal.pone.0139991 (PMC4603877; doi:10.1371/journal.pone.0139991)
Supplement: S3 Table — Arranged according to the abundance. (DOC) [file pone.0139991.s004.doc]

Supporting Information

S3 Table The abundances of phylum (bacterial count > 200) in the two samples. Arranged according to the abundance.

| Phylum | Abundance  (Sample A) |  | Phylum | Abundance  (Sample O) |
| --- | --- | --- | --- | --- |
| *Proteobacteria* | 30.40% |  | *Proteobacteria* | 33.20% |
| *Chloroflexi* | 28.62% |  | *Planctomycetes* | 32.83% |
| *Firmicutes* | 14.75% |  | *Actinobacteria* | 11.66% |
| *Bacteroidetes* | 5.08% |  | *Acidobacteria* | 5.20% |
| *Actinobacteria* | 5.03% |  | *Nitrospirae* | 4.18% |
| *Planctomycetes* | 3.56% |  | *Chloroflexi* | 3.27% |
| *Synergistetes* | 3.52% |  | *Firmicutes* | 1.71% |
| *TM6* | 1.90% |  | *Gemmatimonadetes* | 1.63% |
| *Candidate_division_TM7* | 0.77% |  | *Armatimonadetes* | 1.43% |
| *Thermotogae* | 0.72% |  | *Cyanobacteria* | 0.95% |
| *Unclassified* | 0.70% |  | *Candidate_division_BRC1* | 0.94% |
| *Candidate_division_BR1* | 0.65% |  | *Chlamydiae* | 0.78% |
| *Armatimonadetes* | 0.60% |  | *Bacteroidetes* | 0.67% |
| *Lentisphaerae* | 0.46% |  |  |  |
| *Candidate_division_OP9* | 0.43% |  |  |  |
