# Supplementary material for: The Analysis of a Microbial Community in the UV/O3-Anaerobic/Aerobic Integrated Process for Petrochemical Nanofiltration Concentrate (NFC) Treatment by 454-Pyrosequencing
Source: PLoS One. 2015 Oct 13;10(10):e0139991. doi: 10.1371/journal.pone.0139991 (PMC4603877; doi:10.1371/journal.pone.0139991)
Supplement: S4 Table — Arranged according to the abundance. (DOC) [file pone.0139991.s005.doc]

Supporting Information

S4 Table The abundances of classes (bacterial count > 200) in the two samples. Arranged according to the abundance.

| Class | Abundance  (Sample A) |  | Class | Abundance  (Sample O) |
| --- | --- | --- | --- | --- |
| *Anaerolineae* | 24.60% |  | *Gammaproteobacteria* | 17.09% |
| *Alphaproteobacteria* | 14.22% |  | *Phycisphaerae* | 16.96% |
| *Gammaproteobacteria* | 7.86% |  | *Planctomycetacia* | 15.59% |
| *Clostridia* | 7.75% |  | *Alphaproteobacteria* | 10.12% |
| *Bacilli* | 6.74% |  | *Actinobacteria* | 8.64% |
| *Betaproteobacteria* | 5.78% |  | *Acidobacteria* | 4.95% |
| *Actinobacteria* | 3.58% |  | *Nitrospira* | 4.17% |
| *Synergistia* | 3.51% |  | *Betaproteobacteria* | 3.23% |
| *Planctomycetacia* | 3.26% |  | *Deltaproteobacteria* | 2.78% |
| *Deltaproteobacteria* | 3.23% |  | *Gemmatimonadetes* | 1.63% |
| *Unclassified* | 1.92% |  | *Armatimonadetes_norank* | 1.43% |
| *vadinHA17* | 1.91% |  | *Thermomicrobia* | 1.43% |
| *TM6_norank* | 1.89% |  | *Clostridia* | 1.25% |
| *Bacteroidia* | 1.70% |  | *Acidimicrobiia* | 1.16% |
| *Caldilineae* | 1.36% |  | *Nitriliruptoria* | 0.96% |
| *Flavobacteria* | 0.94% |  | *Candidate_division_BRC1_norank* | 0.94% |
| *Candidate_division_TM7_norank* | 0.76% |  | *Chlamydiae* | 0.78% |
| *Thermoleophilia* | 0.75% |  | *Thermoleophilia* | 0.73% |
| *Thermotogae* | 0.71% |  | *Unclassified* | 0.73% |
| *Candidate_division_BRC1_norank* | 0.64% |  | *SM1D11* | 0.59% |
| *Armatimonadetes_norank* | 0.60% |  | *Caldilineae* | 0.55% |
| *Thermomicrobia* | 0.57% |  | *JG30-KF-CM66* | 0.53% |
| *Lentisphaeria* | 0.46% |  | *TK10* | 0.50% |
| *Candidate_division_OP9_norank* | 0.43% |  |  |  |
