# Supplementary material for: The Analysis of a Microbial Community in the UV/O3-Anaerobic/Aerobic Integrated Process for Petrochemical Nanofiltration Concentrate (NFC) Treatment by 454-Pyrosequencing
Source: PLoS One. 2015 Oct 13;10(10):e0139991. doi: 10.1371/journal.pone.0139991 (PMC4603877; doi:10.1371/journal.pone.0139991)
Supplement: S5 Table — Arranged according to the abundance. (DOC) [file pone.0139991.s006.doc]

Supporting Information

S5 Table The abundances of genera (bacterial count > 200) in the two samples. Arranged according to the abundance.

| Genus | Abundance  (Sample A) |  | Genus | Abundance  (Sample O) |
| --- | --- | --- | --- | --- |
| *Uncultured* | 29.03% |  | *SM1A02* | 15.32% |
| *Unclassified* | 12.37% |  | *Planctomyces* | 8.77% |
| *Pseudomonas* | 3.24% |  | *uncultured* | 7.20% |
| *Aquabacterium* | 2.99% |  | *Unclassified* | 6.89% |
| *Clostridium* | 2.98% |  | *KCM-B-112_norank* | 5.98% |
| *Solibacillus* | 2.47% |  | *Gordonia* | 5.89% |
| *Planococcaceae_Incertae_Sedis* | 2.24% |  | *uncultured_norank* | 5.14% |
| *Peptostreptococcaceae_Incertae_Sedis* | 2.06% |  | *Nitrospira* | 4.17% |
| *Methylocystis* | 1.96% |  | *Blastocatella* | 3.58% |
| *vadinHA17_norank* | 1.91% |  | *Legionella* | 1.80% |
| *TM6_norank* | 1.89% |  | *Thiobacillus* | 1.56% |
| *Leptolinea* | 1.74% |  | *Urania-1B-19_ marine_ sediment_group* | 1.50% |
| *Pirellula* | 1.44% |  | *Armatimonadetes_norank* | 1.43% |
| *Stenotrophomonas* | 1.42% |  | *Hyphomicrobium* | 1.43% |
| *Acinetobacter* | 1.41% |  | *Thioalkalivibrio* | 1.37% |
| *Longilinea* | 1.31% |  | *MSB-1E8_norank* | 1.16% |
| *Flavobacterium* | 0.93% |  | *GR-WP33-30_norank* | 1.15% |
| *Hyphomicrobium* | 0.87% |  | *Nitriliruptor* | 0.95% |
| *Sporosarcina* | 0.85% |  | *Candidate_division_BRC1_norank* | 0.94% |
| *vadinBC27_wastewater-sludge_group* | 0.85% |  | *Sh765B-TzT-29_norank* | 0.91% |
| *Planomicrobium* | 0.84% |  | *Pir4_lineage* | 0.88% |
| *Hydrogenophaga* | 0.81% |  | *Mycobacterium* | 0.73% |
| *BD1-7_clade* | 0.77% |  | *AKYH478_norank* | 0.71% |
| *Candidate_division_TM7_norank* | 0.77% |  | *Candidatus_Alysiosphaera* | 0.65% |
| *Aminobacterium* | 0.68% |  | *Chlamydiaceae_norank* | 0.65% |
| *Candidate_division_BRC1_norank* | 0.65% |  | *Subgroup_6_norank* | 0.61% |
| *Armatimonadetes_norank* | 0.61% |  | *Dietzia* | 0.60% |
| *MNG7_norank* | 0.61% |  | *Sphaerobacter* | 0.57% |
| *Syntrophomonas* | 0.54% |  | *JG30-KF-CM66_norank* | 0.53% |
| *PeM15_norank* | 0.54% |  | *Nocardia* | 0.52% |
| *Planctomyces* | 0.47% |  | *TK10_norank* | 0.50% |
| *480-2_norank* | 0.46% |  |  |  |
| *Candidate_division_OP9_norank* | 0.43% |  |  |  |
| *Afipia* | 0.41% |  |  |  |
