# Supplementary material for: The Analysis of a Microbial Community in the UV/O3-Anaerobic/Aerobic Integrated Process for Petrochemical Nanofiltration Concentrate (NFC) Treatment by 454-Pyrosequencing
Source: PLoS One. 2015 Oct 13;10(10):e0139991. doi: 10.1371/journal.pone.0139991 (PMC4603877; doi:10.1371/journal.pone.0139991)
Supplement: S6 Table — Arranged according to the abundance. (DOC) [file pone.0139991.s007.doc]

Supporting Information

S6 Table The abundances of species (bacterial count > 200) in the two samples. Arranged according to the abundance.

| Species | Abundance  (Sample A) |  | Species | Abundance  (Sample O) |
| --- | --- | --- | --- | --- |
| *Unclassified* | 42.15% |  | *Unclassified* | 40.76% |
| *uncultured_Longilinea_sp.* | 7.07% |  | *uncultured_bacterium* | 16.78% |
| *uncultured_Banisveld_landfill_bacterium* | 5.30% |  | *uncultured_Planctomycetales_bacterium* | 12.93% |
| *Solibacillus_silvestris_StLB046* | 2.47% |  | *Gordonia_rubripertincta* | 5.89% |
| *Pseudomonas_stutzeri* | 2.27% |  | *uncultured_soil_bacterium* | 4.30% |
| *Psychrobacillus_psychrodurans* | 2.24% |  | *uncultured_alpha_proteobacterium* | 1.93% |
| *Clostridium_bowmanii* | 1.71% |  | *uncultured_proteobacterium* | 1.59% |
| *uncultured_Bacteroidetes_bacterium* | 1.70% |  | *uncultured_organism* | 1.36% |
| *Acinetobacter_lwoffii* | 1.13% |  | *uncultured_Chloroflexi_ bacterium* | 1.13% |
| *Pseudomonas_geniculata* | 1.06% |  | *uncultured_candidate_division_SBR1093_bacterium* | 0.90% |
| *[Clostridium]_bifermentans* | 0.83% |  | *uncultured_gamma_proteobacterium* | 0.75% |
| *uncultured_Chloroflexi_bacterium* | 0.81% |  | *Nocardia_cyriacigeorgica x* | 0.52% |
| *Pseudomonas_fulva* | 0.77% |  | *Dietzia_maris* | 0.46% |
| *Uncultured Peptostreptococcaceae_bacterium* | 0.76% |  | *uncultured_Actinomycetales_bacterium* | 0.37% |
| *uncultured_Pelospora_sp.* | 0.48% |  | *Mycobacterium_parascrofulaceum* | 0.34% |
| *uncultured_Planctomycetac* | 0.45% |  | *uncultured_Verrucomicrobia_bacterium* | 0.28% |
| *Synergistetes_bacterium_enrichment* | 0.43% |  | *uncultured_actinobacterium* | 0.26% |
| *Microbacterium_oxydans* | 0.41% |  | *uncultured_sediment_bacterium* | 0.26% |
| *uncultured_Firmicutes_bacterium* | 0.37% |  | *uncultured_Burkholderiaceae_bacterium* | 0.25% |
| *Stenotrophomonas_nitritireducens* | 0.36% |  |  |  |
| *uncultured_Devosia_sp.* | 0.31% |  |  |  |
| *uncultured_Hyphomicrobium_sp.* | 0.30% |  |  |  |
| *uncultured_actinobacterium* | 0.27% |  |  |  |
| *uncultured_Nocardioides_sp.* | 0.25% |  |  |  |
| *Clostridium_butyricum* | 0.23% |  |  |  |
| *Desulfococcus_sp._DSM*  *_8541* | 0.22% |  |  |  |
| *Clostridium_sp._6-69* | 0.20% |  |  |  |
